# NEUROFILAMENT LIGHT CHAIN (NFL) AND COGNITIVE PERFORMANCE INCONSISTENCY IN ADULTS APPROACHING MID LIFE

**DOI:** 10.1093/geroni/igad104.0874

**Published:** 2023-12-21

**Authors:** Chandra Reynolds, Elizabeth Muñoz, Donald Evans, Andrew Smolen

**Affiliations:** University of Colorado Boulder, Boulder, Colorado, United States; The University of Texas at Austin, Austin, Texas, United States; University of Colorado - Boulder, Boulder, Colorado, United States; University of Colorado Boulder, Boulder, Colorado, United States

## Abstract

Neurofilament light chain (NfL) indexes axonal integrity, where higher NfL values correlate with lower cognitive performance. However, associations of NfL with variability in performance and earlier in the lifespan are unclear. We evaluated inconsistency in trial-level performance within three speed or spatial tasks and dispersion across nine verbal, spatial, speed, and episodic memory tasks in participants from the Colorado Adoption/Twin Study of Lifespan behavioral development and cognitive aging (CATSLife1). Individuals were tested at 28-49 years (M=33.1, SD=4.9). Quanterix Simoa assays of plasma NfL (pNfL, log-transformed), cognitive test scores, and sociodemographic covariates were available for up to 1144 individuals. Multi-level regression analyses accounted for sibling relatedness and sociodemographic covariates. Above average pNfL coupled with higher age were associated with greater inconsistency across trials for the WAIS-III Digit Symbol subtest (d=.15 per 3 years of age increase, p=0.037) adjusting for performance, and with reduced performance across age (d=-.27, p=0.014). Moreover, higher pNfL was associated with higher dispersion among nine specific cognitive ability tasks across age, adjusting for general cognitive ability (GCA) factor scores (d=.156, p=0.037). Building on our earlier report of plasma NfL and GCA, higher pNfL was associated with greater dispersion in performance across a battery of tests. Moreover, higher pNfL was associated with poorer overall speed performance, which was also observed for trial level inconsistency even when adjusting for performance level. Hence, at equal levels of ability, within-task inconsistency and dispersion across tasks may be associated with plasma neurofilament light chain in early- to mid-adulthood.

